# Improving Water Use Efficiency of Lettuce (*Lactuca sativa* L.) Using Phosphorous Fertilizers

**DOI:** 10.1186/2193-1801-2-563

**Published:** 2013-10-25

**Authors:** Asad M F AlKhader, Azmi M Abu Rayyan

**Affiliations:** Horticulture and Field Crops, National Center for Agricultural Research and Extension (NCARE), PO Box (639), Baqa’, 19381 Jordan; Faculty of Agriculture, University of Jordan (UOJ), Amman, Jordan

**Keywords:** Lettuce, *Lactuca sativa* L, Phosphorous, Fresh and dry weights, Water use efficiency, Phosphate rock, Alkaline, Calcareous soil

## Abstract

A greenhouse pot experiment was conducted to evaluate the effect of phosphorous (P) fertilizers application to an alkaline calcareous soil on the water use efficiency (WUE) of lettuce cultivar “robinson” of iceberg type. Head fresh and dry weights, total water applied and WUE were affected significantly by the P fertilizer type and rate. P fertilizers addition induced a significant enhancement in the WUE and fresh and dry weights of the crop. A local phosphate rock (PR) applied directly was found to be inferior to the other types of P fertilizers (Mono ammonium phosphate (MAP), Single superphosphate (SSP), and Di ammonium phosphate ((DAP)). MAP fertilizer at 375 and 500 kg P_2_O_5_/ha application rates recorded the highest significant values of head fresh weight and WUE, respectively.

## Introduction

Globally, the paucity of water resources limits agricultural production. The increasing demand for food and water necessitates a more efficient water use of water in agriculture. Jordan is considered one of the ten poorest countries in water resources in the world (Al-Qerem, et al. 2012). Irrigation accounts for 62 % of the total water use in the country in the year 2005, and the allocated water for irrigation in the year 2003 was 511 million cubic meters (Ministry of Water and Irrigation 2004). Improved water use efficiency (WUE) represents a key factor in increasing crop productivity under such water scarcity conditions. Therefore, scientific research in this context to save irrigation water and improve its productivity in Jordan is extremely needed.

Phosphorus (P), in a balanced nutrient management program, can improve WUE and helps crops achieve optimal performance under limited moisture conditions (Briggs and Shantz 1913; Power et al. 1961). It was indicated that increasing P supply had a positive effect of on crop production and WUE (Pyne et al. 1992). Water use efficiency can be expressed as units of yield per unit of water used. Researchers (Ogata et al. 1960) had reported that the considerable enhancement in the water use and WUE by the crop could be attributed to the increase in root growth with high P supply.

Phosphorus is highly needed to establish and maintain crops especially in calcareous soils where the availability of P is very low (Siam et al. 2008). P-deficient plants are known to have lower photosynthetic rates, and decreased growth (Jacob and Lawlor 1991). However, adequately fertilized soils promote rapid leaf area expansion, thus increasing transpiration, and more rapid ground cover, which in turn reduces evaporation and increases WUE. Such increases have been largely attributed to a larger ratio of transpiration to evapotranspiration as a result of greater leaf area (Schmidhalter and Studer 1998).

Phosphate rock (PR) has been used directly in the world, especially in acid soils, as a supplemental P source at different levels but much less than other water-soluble P fertilizers (Khasawneh and Doll 1978). As P is an essential element for its growth and development, lettuce P demand is very high (Lana et al. 2004; Hasaneen et al. 2009). Therefore, lettuce can be used as a test plant.

The objective of this study was to investigate the performance of the lettuce head plant under varying types and rates of P fertilizers application in terms of fresh weight and WUE.

## Materials and methods

### Experimental site

A greenhouse pot experiment was conducted during the growing season 2009/2010 in The Jubeiha Agricultural Research Station of the University of Jordan in the University Campus which is located at 32° 40“ North and 35° 52” East, and 980 m above sea level and has a mean annual rainfall of about 414 mm.

### Plant material

Seedlings of lettuce (*Lactuca sativa* L.) cultivar “robinson” of iceberg type of 35 days after sowing (DAS) were obtained from a commercial nursery.

### Experimental design and treatments

**Figure 1:**
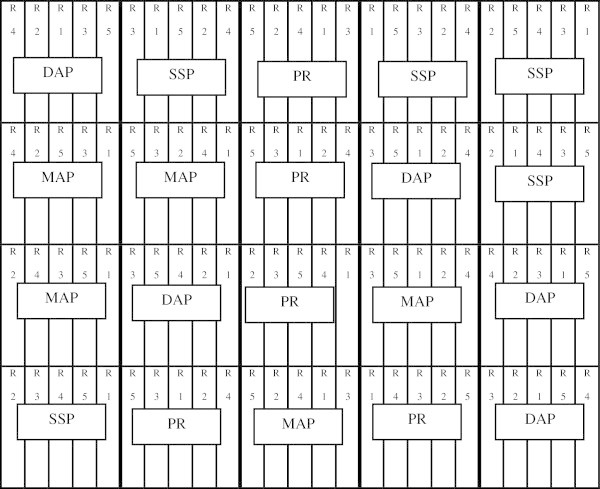
**Experimental design layout of the greenhouse pot experiment using a split plot arrangement in a completely randomized design (CRD); the main plot treatments consist of 4 P fertilizers types (Single super phosphate (SSP), Di ammonium phosphate (DAP), Mono ammonium phosphate (MAP) and a local Phosphate rock (PR)); the subplot treatments consist of 5 P rates (R1 = 0, R2 = 125, R3 = 250, R4 = 375, and R5 = 500 kg P**_**2**_ **O**_**5**_ **/ha) with 5 replicates.**

The experimental design used was a split-plot arrangement in a completely randomized design (CRD) (Figure 1). Where four fertilizers types (Single super phosphate (SSP), Di ammonium phosphate (DAP), Mono ammonium phosphate (MAP) and a local phosphate rock (PR) in a finely ground form (powder)) were assigned to the main plots, while five fertilizers rates (R1 = 0, R2 = 125, R3 = 250, R4 = 375, and R5 = 500 kg P_2_O_5_/ha) were assigned to the subplots, replicated five times.

### Cultural practices

**Figure 2:**
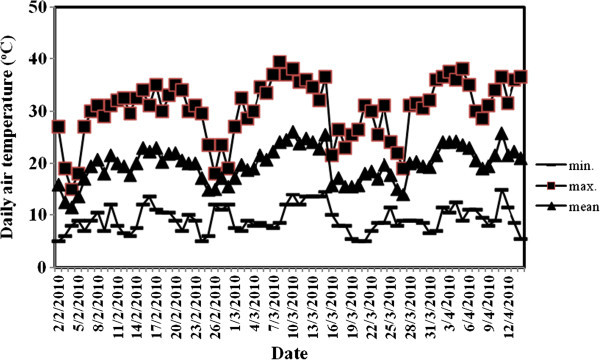
**Minimum, maximum and mean daily air temperature for selected days in the greenhouse experiment during the growing season 2009/2010 at The Jubeiha Agricultural Research Station of the University of Jordan.**

**Figure 3:**
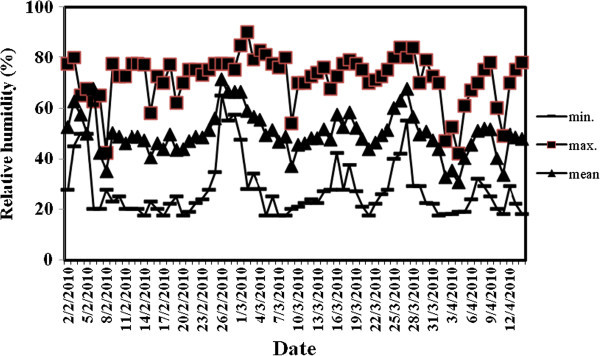
**Minimum, maximum and mean daily relative humidity for selected days in the greenhouse experiment during the growing season 2009/2010 at The Jubeiha Agricultural Research Station of the University of Jordan.**

**Figure 4:**
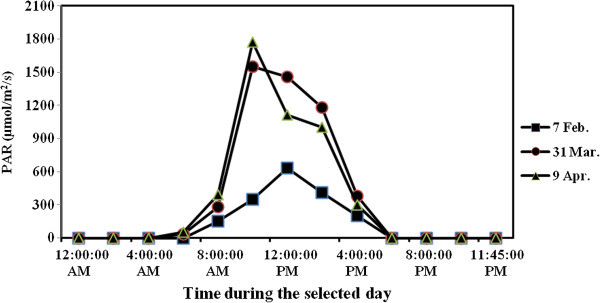
**Photosynthetic active radiation (PAR) measured inside the greenhouse during three selected days of three months (7 Febraury, 31 March and 9 April 2010) during the growing season 2009/2010 at The Jubeiha Agricultural Research Station of the University of Jordan.**

**Figure 5:**
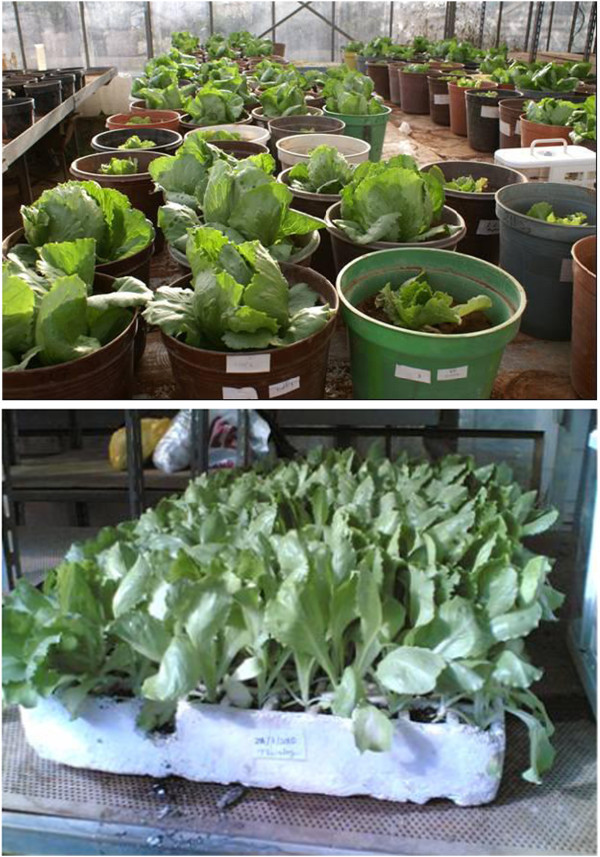
**The greenhouse pot experiment 36 days after transplanting (DAT) which carried out on 2/2/2010 (top), and the seedlings of the lettuce cultivar “robinson” of iceberg type (35 days after sowing (DAS)) used in the transplantation (bottom).**

Plastic pots of 7 liter size, 25 cm top diameter, and 26 cm height were used. Air dry clay texture soil (clay 47.7%, silt 36.8% and sand 15.5%) of 7 Kg weight was put in each pot after being mixed, and fumigated with methyl bromide. P fertilizers and urea (46% N) at a rate of 200 Kg N/ha were thoroughly mixed with the soil of each pot. Supplemental N was applied to make sure that each treatment receives the same level of N. Lettuce seedlings were planted on 2^nd^ February 2010 with one transplant per pot. Soil moisture was maintained close to field capacity during the growing season. Daily air temperature (Figure 2) and relative humidity (RH) (Figure 3) inside the greenhouse were monitored using a thermohigrograph (Thies, CLIMA, Germany) where the chart was replaced weekly. Photosynthetic active radiation (PAR), also, was measured using Mini station (WatchDog, Spectrum Technologies, Inc.), as shown in Figure 4. Harvesting was carried out on 4^th^ April 2010 (61 days after transplanting, (DAT)). Figure 5 shows the greenhouse pot experiment 36 DAT and the seedlings used in the transplantation.

### Chemical and physical analysis

#### Soil

Soil samples were air dried, crushed and passed through a 2 mm sieve for some chemical and physical analysis. Soil pH and salinity as paste extract, cation exchange capacity (CEC), texture (hydrometer method), organic matter, calcium carbonate (calcimeter method), total N (Kjeldhal method), available P (using spectrophotometer), available K (using flame photometer) were determined according to the previous procedures, respectively (Bower and Wilcox 1965; Chapman 1965; Day 1965; Allison 1965; Allison and Moodie 1965; Bremner 1965; Olsen and Dean 1965; Pratt 1965). The results of the analysis are presented in Table 1.

**Table 1:** **Results of some chemical and physical properties of the soil used in the pot experiment**

Texture	CEC	Available		O. M.	Total N	CaCO_3_	Salinity EC	pH
		P	K				dS/m	
	meq/100 g	ppm		%			Paste extract	
Clay	43.4	6.1	313	1.58	0.08	8.2	0.96	7.75

#### Irrigation water

Chemical analysis for the irrigation water used in the experiment was conducted to determine pH, electrical conductivity (EC), and major cations and anions according to the previous described work (Chapman and Pratt 1982). Table 2 shows the results of the analysis of the irrigation water.

**Table 2:** **Results of chemical analysis for the irrigation water used in the pot experiment**

NO_3_^-^	SO_4_^2-^	HCO_3_^-^	Cl^-^	K^+^	Na^+^	Mg^2+^	Ca^2+^	EC	pH
ppm	meq/l							dS/m	
3.74	3.55	0.9	5.5	0.15	4.26	2.6	2.94	1.03	7.8

#### Plant

##### Head fresh weight

The head fresh weight was determined using an electronic balance (± 0.1 g).

##### Dry matter

Plant samples (leaves and stems) were dried in the oven at 65°C for 72 hrs and the dry matter was determined (± 0.1 g).

### Statistical analysis

Analysis of variance (ANOVA) and mean separation according to least significant difference (LSD) at the 5% level of significance were conducted for the results using SAS version 9.0 (SAS Institute Inc. 2002).

## Results and discussion

### Fresh and dry weights

**Figure 6:**
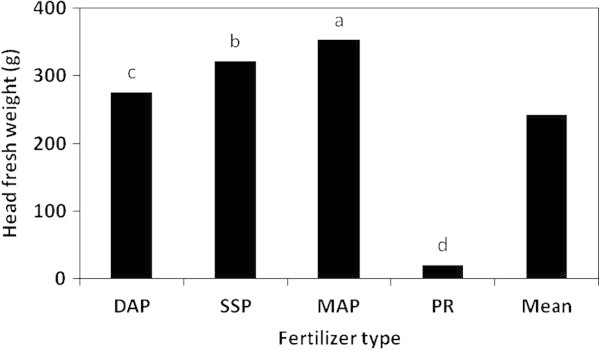
**The effect of the P fertilizer type on the head fresh weight of the lettuce plant in the greenhouse pot experiment.**

**Figure 7:**
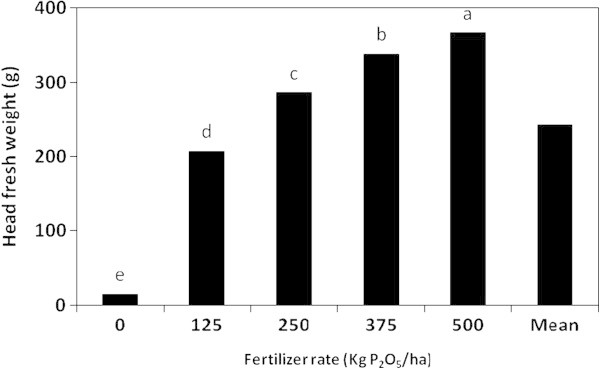
**The effect of the P fetilizer rate on the head fresh weight of the lettuce plant in the greenhouse pot experiment.**

The results indicated that fresh and dry weights of the lettuce head plant were significantly affected by the P fertilizer type, rate and their interaction (Table 3). Plants fertilized with MAP produced the highest fresh and dry weights/head (353.4 and 14.13 g/head, respectively), whereas the lowest weights were related to PR-fertilized plants (20.2 and 0.81 g/head, respectively) (Figure 6). MAP, SSP and DAP induced significant differences in plant fresh and dry weights/head as follows: MAP > SSP > DAP > PR. On the other hand, the effect of the P rate on the head fresh and dry weights was in the following descending order: 500 < 375 < 250 < 125 < 0 kg P_2_O_5_/ha kg P_2_O_5_/ha. The highest fresh and dry weights/head were linked to the rate of 500 kg P_2_O_5_/ha (366.7 and 4.66 g/head, respectively), whereas the control treatment (zero P) recorded the lowest weights (14.8 and 0.58 g/head, respectively) (Figure 7).

**Table 3:** **Effect of P fertilizer on lettuce plant growth, total water applied and water use efficiency**

Fertilizer type	Fresh weight	Dry weight	Total applied water	Water use efficiency
	g/head		mm	g/mm
DAP	275.9 c	11.03 c	160.1 b	1.61 b
SSP	321.0 b	12.83 b	172.5 ab	1.75 ab
MAP	353.4 a	14.13 a	178.7 a	1.87 a
PR	20.2 d	0.81 d	115.7 c	0.17 c
Mean	242.6	9.70	156.7	1.35
LSD _0.05_	22.7	0.91	14.6	0.16
**Fertilizer rate (kg P**_**2**_ **O**_**5**_ **/ha)**				
0	14.8 e	0.58 e	112.0 c	0.13 c
125	207.0 d	8.28 d	145.1 b	1.35 b
250	286.3 c	11.45 c	155.3 b	1.73 a
375	338.3 b	13.53 b	178.6 a	1.70 a
500	366.7 a	14.66 a	192.8 a	1.85 a
Mean	242.6	9.7	156.7	1.35
LSD _0.05_	25.4	1.01	16.3	0.18
**Significance level**				
Fertilizer type	***	***	***	***
Fertilizer rate	***	***	***	***
Fertilizer type × rate	***	***	**	***

Means followed by different letter(s) in a column differ significantly according to LSD test at 0.05 probability.

**: Highly Significant at P ≤ 0.01.

***: Highly Significant at P ≤ 0.001.

Generally, the highest weights were recorded for the higher application rates of DAP, SSP and MAP, whereas the control treatments and different rates of PR recorded the lowest results (Table 4). There was no significant differences in plant fresh and dry weights among the rates of 500 and 375 kg P_2_O_5_/ha of MAP and 500 kg P_2_O_5_/ha of SSP. However, the maximum fresh and dry weights (531.4 and 21.26 g/head, respectively) were induced at 375 P_2_O_5_/ha fertilization rate of MAP. Meanwhile, the minimum values were reported for the control treatment of PR (13.0 and 0.52 g/head, respectively). No significant increases in the fresh and dry weights were detected as higher P rates had been applied. Thus, the 375 P_2_O_5_/ha rate of MAP can be recommended under similar environments. This would reduce the costs of P application and conserve the natural reserves of phosphate. Positive environmental consequences through minimizing pollution of the environment (Boutraa 2009), also, should be expected.

**Table 4:** **Interaction effect of P fertilizer on lettuce plant growth, water applied and water use efficiency**

Fertilizer type	Fertilizer rate	Fresh weight	Dry weight	Total water applied	Water use efficiency
	(kg P_2_O_5_/ha)	g/head		mm	g/mm
DAP	0	14.1 h	0.56 h	109.8 h	0.13 f
	125	233.7 g	9.35 g	142.5 fg	1.66 e
	250	336.4 ed	13.45 ed	161.5 ef	2.10 bcd
	375	367.9 cd	14.72 cd	183.4 bcde	2.02 6cd
	500	427.0 b	17.08 b	203.2 bc	2.12 bcd
SSP	0	16.8 h	0.67 h	115.1 gh	0.14 f
	125	266.0 fg	10.64 fg	165.3 ef	1.62 e
	250	399.3 bc	15.97 bc	169.0 def	2.38 abc
	375	427.3 b	17.09 b	201.3 bcd	2.21 bcd
	500	495.0 a	19.80 a	212.1 ab	2.42 ab
MAP	0	14.4 h	0.58 h	115.5 gh	0.13 f
	125	306.3 ef	12.25 ef	161.0 ef	1.91 de
	250	388.0 bc	15.52 bc	173.0 cdef	2.26 bcd
	375	531.4 a	21.26 a	205.0 bc	2.34 bc
	500	526.5 a	21.06 a	239.0 a	2.70 a
PR	0	13.0 h	0.52 h	107.8 h	0.12 f
	125	22.1 h	0.89 h	111.7 gh	0.20 f
	250	21.3 h	0.85 h	117.8 gh	0.18 f
	375	26.4 h	1.06 h	124.6 gh	0.21 f
	500	17.8 h	0.71 h	116.7 gh	0.15 f
LSD		50.7	2.03	32.7	0.36

Means followed by different letter(s) in a column differ significantly according to LSD test at 0.05 probability.

The superiority of MAP, SSP and DAP over PR could be attributed to their higher solubility and, thus, higher P availability to the plant as they are fast-release fertilizers (Miretzky and Fernandez 2008; Siam et al. 2008). The results, also, agree with the findings of many researchers (Chien and Menon 1995; Prochnow et al. 2006; Miretzky and Fernandez 2008) which indicated that PRs are of low solubility and, hence, low agronomic efficiency in high pH calcareous soils. Besides their higher solubility, ammonium phosphate fertilizers, like MAP and DAP, are superior to calcium phosphate fertilizers (like PRs) due to the presence of ammonium ion that has a positive effect on plant growth (Beaton and Nielsen 1959).

On the other hand, the relatively high agronomic performance for MAP compared with the other P fertilizers sources can be attributed to the higher production of H_2_PO_4_^-^ which is more readily available to the plants than the other P forms (Fixen 1990).

The enhancement effect of P application on plant growth could be related to the vital role of inorganic P, in the ATP form, which provides energy for CO_2_ assimilation in the Calvin Cycle in plant photosynthesis and the synthesis of starch, fatty acids and amino acids (Mikulska et al. 1998; Luo et al. 2009). However, the reduction of fresh and dry weights of the lettuce plant under lower P application rates and control treatments could be related to the role of the abscisic acid in growth inhibition as its content in plant leaves increases under such suboptimal growth conditions (Mikulska et al. 1998).

### Water applied

**Figure 8:**
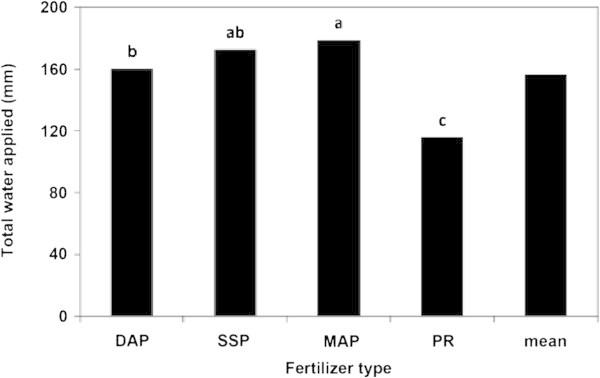
**The effect of P fertilizer type on the total water applied to the lettuce head plant in the greenhouse pot experiment.**

**Figure 9:**
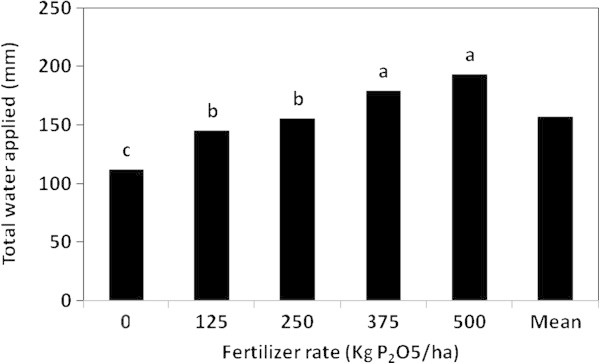
**The effect of P fertilizer rate on the total water applied to the head lettuce plant in the greenhouse pot experiment.**

P fertilizer type, rate and their interaction affected the total amount of water applied to the head lettuce plant significantly (Table 3). As shown in Figure 8, plants fertilized with MAP gained the highest amount of water applied (178.67 mm), meanwhile the lowest amount of water applied was recorded for plants fertilized with PR (115.69 mm). While the application rate of 500 kg P_2_O_5_/ha induced the highest total water applied (192.755 mm), the control treatment caused the lowest amount (112.0 mm), as presented in Figure 9. Actually, the total water applied of the control treatment was significantly lower than those of the other P application rates. However, there were no significant differences between the total water applied at 500 and 375 kg P_2_O_5_/ha rates.

Higher application rates of different fertilizers, except PR, resulted in higher values of water applied (Table 4). This was supported by many investigators (Xu et al. 2004) who indicated that the rate of water uptake was higher at the high P application rate treatment than that at the low one, and this was attributed to the greater size of the plants at the high P level. On the other hand, plants fertilized with MAP at the application rate of 500 kg P_2_O_5_/ha recorded the highest significant value of total water applied (239.03 mm), and the lowest value was obtained at the control treatment of PR (107.77 mm). No significant differences in the total water applied were observed between that recorded at the 500 kg P_2_O_5_/ha rate of each of MAP and SSP fertilizers.

### Water use efficiency

**Figure 10:**
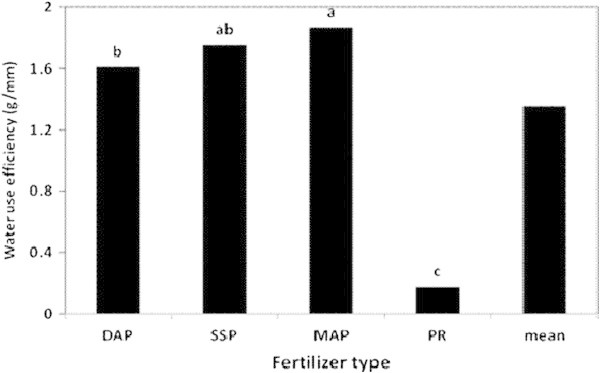
**The effect of P fertilizer type on the water use efficiency by the lettuce head plant in the greenhouse pot experiment.**

**Figure 11:**
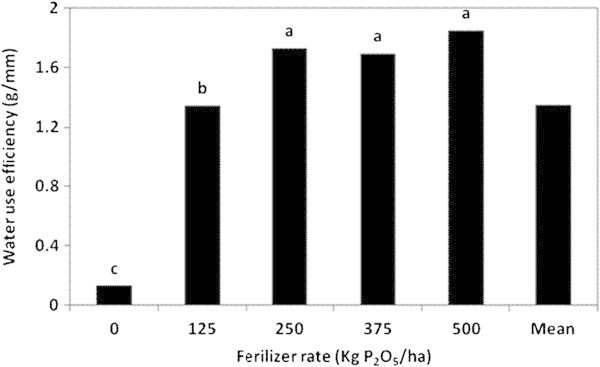
**The effect of P fertilizer rate on the water use efficiency by the head lettuce plant in the greenhouse pot experiment.**

The effects of the fertilizer type, rate and their interaction on the WUE of the lettuce head plant were highly significant (Table 3). While, plants fertilized with MAP presented the highest W.U.E. (1.87 g/mm), the lowest value was reported for plants fertilized with PR (0.17 g/mm). However, there was no significant difference in W.U.E. induced by SSP and MAP fertilizers (Figure 10). On the other hand, the application rates of 250, 375 and 500 kg P_2_O_5_/ha caused no significant differences in the W.U.E. of the plant (1.85, 1.70 and 1.73 g/mm, respectively), as displayed in Figure 11. The control treatment, however, marked the lowest WUE (0.13 g/mm). Table 4 indicates that plants fertilized with MAP at 500 kg P_2_O_5_/ha exhibited the highest W.U.E. (2.70 g/mm), whereas the lowest value of W.U.E. was recorded for plants related to the control treatment of PR (0.12 g/mm). However, the difference in the WUE produced by MAP and SSP fertilizers at 500 kg P_2_O_5_/ha application rate was not significant. The substantial improvement in the WUE by the plant could be ascribed to the increase in the plant root growth with increasing P supply (Ogata et al. 1960).

## Conclusions and recommendations

The investigated P fertilizers, except PR, enhanced the performance of lettuce head plant grown in an alkaline calcareous soil through improving its WUE and increasing its fresh weight and, subsequently, the yield. MAP and SSP fertilizers were found to be superior to the other P fertilizers, and can be used successfully to improve the crop WUE and increase its yield. Direct application of PR to the alkaline calcareous soil was of low agronomic value. MAP fertilizer at the application rate of 375 kg P_2_O_5_/ha can be recommended in terms of both plant fresh weight and WUE, as this treatment of fertilizer can induce both relatively high crop yield and improve irrigation water productivity.
